# Supplementary figures and images for: Assessing the biomedical applicability of biogenically synthesized AuNPs using Salvia splendens extract
Source: PLoS One. 2025 Jun 5;20(6):e0324445. doi: 10.1371/journal.pone.0324445 (PMC12140272; doi:10.1371/journal.pone.0324445)

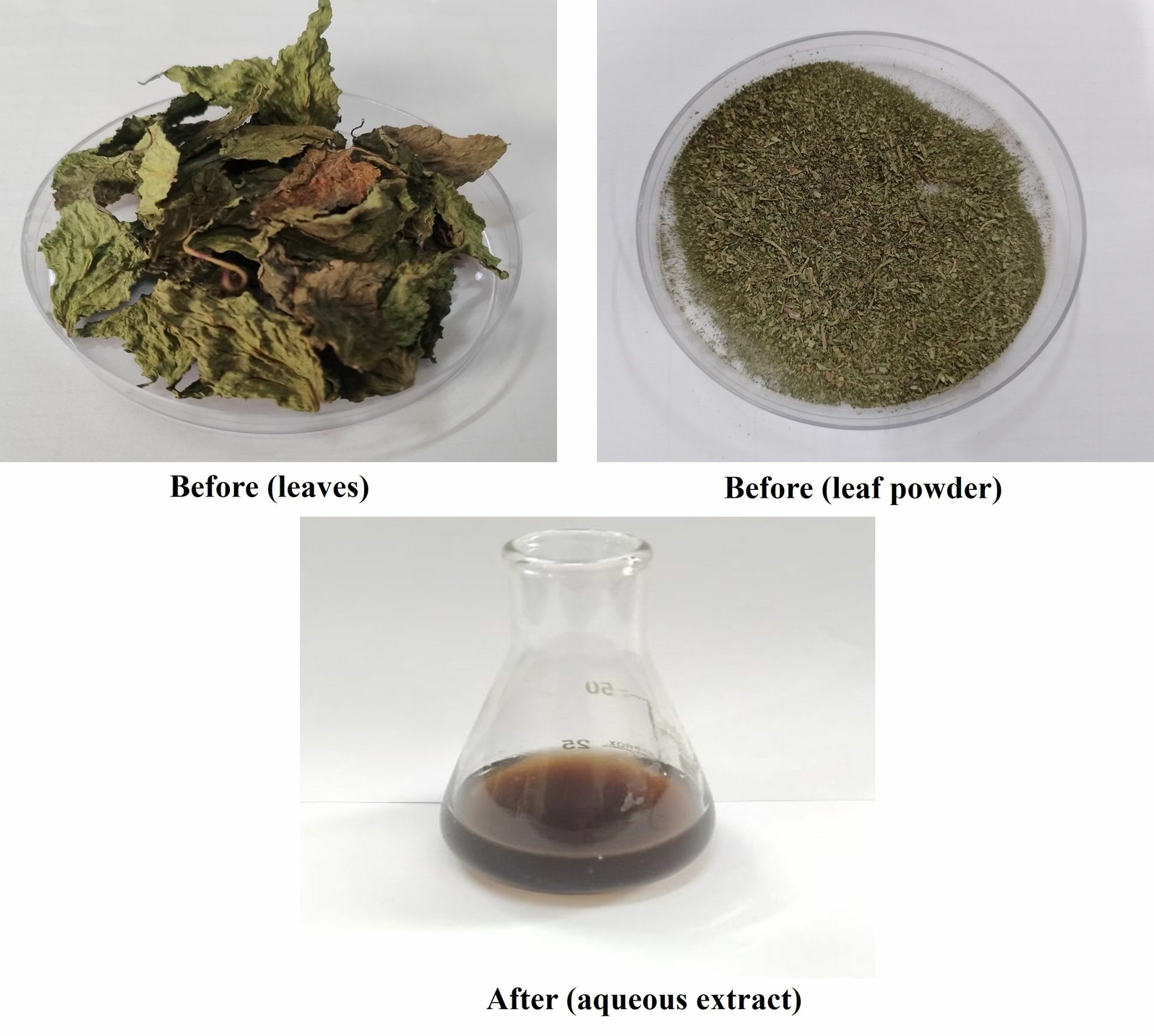

Supplement: S1 Fig — (TIF) [file pone.0324445.s001.tif]
