# Supplementary material for: Assessing the biomedical applicability of biogenically synthesized AuNPs using Salvia splendens extract
Source: PLoS One. 2025 Jun 5;20(6):e0324445. doi: 10.1371/journal.pone.0324445 (PMC12140272; doi:10.1371/journal.pone.0324445)
Supplement: Attachment — Submitted filename: Comments Received D1.docx [file pone.0324445.s003.docx]

**Reviewer 1**

I reviewed this paper; Therefore, I cannot recommend this manuscript to be published unless the authors answer all comments. It needs "major revisions" before acceptance for publication.

**Comment 1:** **Synthesis green mechanism of Au-NPs must be added to text.**

***Authors response: The authors have incorporated the green mechanism of Au-NPs in the revised manuscript from line number 129–135. The same has been highlighted for the kind perusal of learned reviewer.***

**Comment 2:** **FTIR spectra of Au-NPs and Salvia splendens extract must be added to text.**

***Authors response: As per the insightful suggestion of learned reviewer, the authors have performed FTIR of SSLE and SSLE-AuNPs. The same has been incorporated in the material and methods (2.2.4.3) and results section 3.2 in the revised manuscript. The same has been highlighted for the kind perusal of learned reviewer.***

**Comment 3:** **PSA curve of Au-NPs must be added to text according to TEM image.**

***Authors response: As per the suggestion of learned reviewer authors have included the PSA curve of AuNPs in the revised manuscript as figure 1e.***

**Comment 4:** **XRD pattern of Au-NPs must be added to text.**

***Authors response: We sincerely appreciate the reviewer's insightful comment regarding the inclusion of XRD analysis for the biosynthesized SSLE-AuNPs. While XRD is indeed a valuable technique for crystallographic characterization, our current study prioritized complementary methods (UV-Vis, TEM, DLS, and zeta potential) to comprehensively evaluate the optical, morphological, colloidal stability, and size distribution properties of the nanoparticles. These techniques collectively confirmed the successful synthesis of spherical, stable AuNPs with a characteristic SPR peak (559 nm), uniform size distribution (94.8 ± 5.1 nm via TEM), and high colloidal stability (−21 ± 1.9 mV zeta potential).***

***The omission of XRD does not detract from the key conclusions of our study, as the crystallinity of AuNPs is well-documented in literature for similar plant-mediated syntheses, and our TEM analysis indirectly supports crystalline structure. However, we acknowledge the importance of XRD for future work and will include this analysis in subsequent studies to further validate crystallinity and phase purity.***

***The multifunctional biological activities of SSLE-AuNPs—demonstrated through rigorous antioxidant, antibacterial, anticancer, and anti-inflammatory assays—remain robustly supported by the existing dataset. At this point of time, we lack the facilities of XRD at our institute, and we do not have enough funds to outsource XRD analysis. We hope the reviewer agrees that the current findings provide a strong foundation for the therapeutic potential of SSLE-AuNPs, even in the absence of XRD data.***

**Comment 5:** **Last paragraph of the introduction is not appropriately written. Explain the main objective of this work.**

***Authors response: The authors have modified the last paragraph of the introduction section in the revised manuscript line number 142–151 as per the suggestion of learned reviewer. The same is highlighted for valued reference of the learned reviewer.***

**Comment 6:** **Overall, the discussions are not enough and the different sections must be described scientifically.**

***Authors response: We sincerely appreciate the reviewer’s constructive feedback regarding the depth of scientific discussion in our manuscript. We have duly added the discussion section with more rigorous and detailed interpretation of the results, and highlighted it in the revised MS.***

**Comment 7:** **Authors should be appropriate to carefully check the compliance of the article with the English grammar rules by an academic whose native language is English. The English of the entire article should be improved. With more caution, many mistakes can be avoided.**

***Authors response: As per the insightful suggestion of learned reviewer, the authors have checked the compliance of English grammar rules via native English-speaking academician.***

**Comment 8:** **References should be reviewed, and referring to more up-to-date references would be better.**

***Authors response: The authors have updated the references in the revised manuscript as per the suggestion of the learned reviewer. The same are highlighted in the bibliography for kind perusal of the reviewer.***

**Comment 9:** **The abbreviation should be mentioned completely for the first time and mentioned after that as an abbreviation. Please revise throughout the manuscript.**

***Authors response: As per the suggestion of the learned reviewer, the authors checked the consistencies of the abbreviation used in the revised manuscript.***

**Comment 10:** **To give a better view to this work, authors are suggested to improve the introduction and other sections using recent and related articles.**

DOI: 10.1016/j.inoche.2024.113399; DOI: 10.1109/TNB.2023.3287805; DOI: 10.1016/j.ceramint.2023.03.234; DOI: 10.1007/s12010-023-04407-y; DOI: 10.1007/s12257-024-00004-w

***Authors response: As per the valuable suggestion of learned reviewer, the authors have modified the introduction and other sections of manuscript by using these references in the revised manuscript. The stated references have been cited at reference number 4, 5, 6, 36, 37 respectively.***

**Reviewer 2**

The authors have studied the in vitro antioxidant, anticancer, and anti-inflammatory activities of biogenically synthesized AuNPs using Salvia splendens extract. The research work is informative and interesting. However, some inadequacy was noted in the manuscript which needs to be addressed for the improvement of the quality of the research article considering the publication criteria of the highly esteemed journal like Plos One.

The comments are as follows:

**Comment 1:** **The introduction seems to be too preliminary and there is no significant literature support for the natural products-based antioxidant, anti-inflammatory, and anticancer activities. In addition, there needs a better description on the bioactive molecules in the plant extract/ similar plant species which could be responsible for their plausible potential.**

***Authors response: As per the insightful suggestion of learned reviewer, authors have modified the introduction section of revised manuscript by incorporating text explaining description of bioactive molecules in the extract of Salvia species which could be responsible for their plausible potential.***

**Comment 2:** **What is the significance of oxidative stress and inflammation in the development and progression of lung cancer? Authors could stress these details in the introduction part.**

***Authors response: The authors would like to state that oxidative stress and inflammation are two interrelated biological processes that play a crucial role in the development and progression of lung cancer. Understanding these mechanisms is essential for elucidating the pathophysiology of lung cancer and identifying potential therapeutic targets.***

***Indeed, it has now been established that chronic prevalence of oxidative stress serves to be a critical driver for lung carcinogenesis along with tumor aggressiveness [1]. Increased reactive oxygen species (ROS) mediated oxidative stress not only triggers the damage of the genetic material but also activates several oncogenes including KRAS and EGFR [2]. Furthermore, increased ROS levels are also associated with imparting resistance towards apoptosis with concomitantly inducing the activation of pro-survival pathways such as PI3K/AKT and NF-κB [3, 4]. At molecular level, inflammation within tumor microenvironment is mediated by macrophages and cytokines. These cytokines predominantly include IL-1β, IL-6 and TNF-α among others which promotes angiogenesis, immune evasion and metastasis [5]. The inflammatory cytokine milieu concomitantly with increased ROS amplifies epithelial mesenchymal transition (EMT) and mutagenesis [6].***

***This interplay creates a vicious cycle where both processes amplify each other, leading to sustained cellular damage, genetic instability, and ultimately contributing significantly to lung carcinogenesis. Furthermore, the authors have incorporated this section in the revised manuscript.***

***1. Bezerra FS, Lanzetti M, Nesi RT, Nagato AC, Silva CP, Kennedy-Feitosa E, Melo AC, Cattani-Cavalieri I, Porto LC, Valenca SS. Oxidative stress and inflammation in acute and chronic lung injuries. Antioxidants. 2023 Feb 21;12(3):548.***

***2. Ahmad I, Ahmad S, Ahmad A, Zughaibi TA, Alhosin M, Tabrez S. Curcumin, its derivatives, and their nanoformulations: Revolutionizing cancer treatment. Cell Biochemistry and Function. 2024 Jan;42(1):e3911***

***3. Ahmad I, Hoque M, Alam SS, Zughaibi TA, Tabrez S. Curcumin and plumbagin synergistically target the PI3K/Akt/mTOR pathway: a prospective role in cancer treatment. International journal of molecular sciences. 2023 Apr 2;24(7):6651.***

***4. Suhail M, Rehan M, Tarique M, Tabrez S, Husain A, Zughaibi TA. Targeting a transcription factor NF-κB by green tea catechins using in silico and in vitro studies in pancreatic cancer. Frontiers in Nutrition. 2023 Jan 11;9:1078642.***

***5. Nigam M, Mishra AP, Deb VK, Dimri DB, Tiwari V, Bungau SG, Bungau AF, Radu AF. Evaluation of the association of chronic inflammation and cancer: Insights and implications. Biomedicine & Pharmacotherapy. 2023 Aug 1;164:115015.***

***6. Tripathi S, Sharma Y, Kumar D. Unveiling the link between chronic inflammation and cancer. Metabolism Open. 2025 Jan 9:100347.***

**Comment 3: Incorporate the machine generated data in the figure 1d instead of incorporating the data manually.**

***Authors response: As per the suggestion of the reviewer, machine generated data has been added in the figure 1d of the revised manuscript.***

**Comment 4:** **Incorporate a size distribution graph in the TEM results.**

***Authors response: As per the suggestion of the learned reviewer, the authors have incorporated size distribution graph in the TEM results of revised manuscript in fig. 1e.***

**Comment 5:** **The authors should explain the basis for keeping treatment duration for 24 h in MTT/ cytotoxicity assay.**

***Authors response: The authors would like to state that duration of treatment in the MTT assay, particularly the common practice of using a 24-hour incubation period, is primarily based on several scientific and practical considerations that ensure reliable and interpretable results regarding cell viability and cytotoxicity which are listed as follows:***

***1. Cells require a certain amount of time to respond to treatments, such as drugs or other cytotoxic agents. A 24-hour period allows sufficient time for the cells to metabolize the treatment and exhibit any potential effects on their metabolic activity. This timeframe is generally considered optimal for observing significant changes in cell viability or metabolic activity without being influenced by acute toxicity that may occur with shorter exposure times.***

***2. The MTT assay measures cellular metabolic activity through the reduction of the MTT reagent to formazan crystals, which occurs predominantly in metabolically active cells. A 24-hour incubation provides enough time for viable cells to reduce MTT effectively, leading to a measurable accumulation of formazan. Shorter durations may not allow adequate time for this reaction to occur, potentially resulting in underestimating cell viability or overestimating cytotoxicity.***

***3. Using a standardized treatment duration like 24 hours facilitates comparability across different studies and experiments. Many researchers adopt this timeframe as it has been widely validated in literature, allowing for consistent interpretation of results and better reproducibility when comparing findings from various laboratories.***

**Comment 6:** **In vivo work would have been strengthening the present findings.**

***Authors response: The authors would like to state that although in vivo studies are crucial for understanding the therapeutic potential and safety profile of novel compounds, the current study focused on establishing the in vitro efficacy and multifunctional potential of biogenically synthesized gold nanoparticles (SSLE-AuNPs) using Salvia splendens leaf extract. The primary objective of this research was to provide a foundational understanding of the antioxidant, antibacterial, anticancer, and anti-inflammatory properties of SSLE-AuNPs in controlled in vitro environments. Furthermore, the authors have limited their current study to in vitro level due to following considerations which are listed as follows:***

***1. This study serves as a preliminary investigation to establish the proof of concept for the multifunctional potential of SSLE-AuNPs. Before progressing to in vivo studies, it is essential to thoroughly characterize the nanoparticles and confirm their biological activities in in vitro models. This step ensures that only the most promising candidates are advanced to more complex and resource-intensive in vivo studies.***

***2. Given the exploratory nature of this research, the focus was first placed on optimizing the synthesis, characterization, and in vitro evaluation of SSLE-AuNPs to ensure their potential before committing to in vivo experimentation. However, in vivo studies require significant resources, including ethical approvals, specialized animal care facilities, and longer experimental timelines.***

***3. The promising results obtained from the in vitro studies provide a strong rationale for future in vivo investigations. Subsequent research will focus on evaluating the pharmacokinetics, biodistribution, toxicity, and therapeutic efficacy of SSLE-AuNPs in appropriate animal models. This stepwise approach ensures a more comprehensive understanding of the nanoparticles' potential before advancing to clinical applications.***

**Comment 7:** **There are numerous published reports demonstrating the potential of synthesized plant-based nanoformulations. Hence, what is the novelty and exclusiveness of this work needs to be explained.**

***Authors response: The authors would like to state that the novelty and exclusiveness of this work lie in multiple key aspects that differentiate it from previously published reports on plant-based nanoformulations:***

***1. Our present study utilizes the leaf extract of Salvia splendens (SSLE) for the biosynthesis of gold nanoparticles (AuNPs). However, there are numerous reports on the use of plant extracts for nanoparticle synthesis, the specific use of Salvia splendens for this purpose is relatively unexplored. The plant is known for its bioactive components, but its application in the green synthesis of AuNPs and the subsequent evaluation of their multifunctional biomedical potential is novel.***

***2, The green synthesized gold nanoparticles in the present investigation can scavenge free radicals (like antioxidant molecules/compounds) in normal condition, contrastingly, to get rid of the cancer cell they can generate significant amount of ROS as well.***

***3. The current study evaluates the multifunctional potential of the biosynthesized SSLE-AuNPs that possess anti-bacterial potential against both gram-negative and -positive strains, anti-cancer potential via modulating caspase-3 activity and activating apoptotic pathway, and anti-inflammatory potential via downregulating proinflammatory cytokines such as IFN-γ and IL-1β. It is observed that various published studies focus only on exploring one or two biological attributes; however, our present investigation provides a holistic assessment of the nanoparticles' biomedical applicability.***

***4. The study provides detailed mechanistic insights into the anticancer activity of SSLE-AuNPs, particularly in lung cancer cells (A549). It explores the induction of apoptosis through nuclear fragmentation, caspase activation, and ROS generation. Such detailed mechanistic studies, especially in the context of lung cancer, are not commonly reported for plant-based nanoformulations.***

***5. The current report specifically targets lung cancer cells (A549) and provides evidence of the cytotoxic effects of SSLE-AuNPs, suggesting their potential as a therapeutic agent for lung cancer. Given the high mortality rate associated with lung cancer, this focus on a specific type of cancer adds to the exclusiveness of the work.***

***These stated points have been rephrased and included in the introduction section of the revised manuscript.***

**Comment 8:** **How can the authors corroborate the antioxidant and anticancer/ cytotoxicity of the extract?**

***Authors response: In response to the concern raised by the learned reviewer the authors would like to state that modulation of intracellular antioxidant concentration is a double-edged sword, with both sides exploited for potential therapeutic benefits. While antioxidants may hamper the efficacy of chemotherapy by scavenging reactive oxygen species and free radicals, it is also possible that antioxidants alleviate unwanted chemotherapy-induced toxicity, thus allowing for increased chemotherapy doses. Under normal environment, antioxidants neutralize toxic oxidants, such as reactive oxygen species (ROS), maintaining them within narrow boundaries level. This redox balance is achieved by various scavenging systems such as enzymatic system (e.g., superoxide dismutases, catalase, and peroxiredoxins), nonenzymatic systems (e.g., glutathione, cysteine, and thioredoxin), and metal-binding proteins (e.g., ferritin, metallothionein, and ceruloplasmin) that sequester prooxidant metals inhibiting their participation in redox reactions. On the other hand, therapeutic strategies that promote oxidative stress and eventually tumor cells apoptosis have been explored based on availability of chemotherapy agents that inhibit ROS-scavenging systems. Therefore, understanding the antioxidant-driven molecular pathways might be crucial to design new therapeutic strategies to fight cancer progression.***

**Comment 9:** **The concentration of DPPH solution (100 mM) used in this study is well beyond the recommended level. It should be in micromole (µM) concentration.**

***Authors response: The authors agree with the learned reviewer that the concentration of DPPH (2,2-diphenyl-1-picrylhydrazyl) solution used in antioxidant assays is typically in the micromolar (µM) range, not millimolar (mM). However, the authors would like to extend their deepest apologies for this unintentional typological error and the correction has been done at line 209 of the revised manuscript.***

**Comment 10:** **The** **absorbance of formazan is a peak at 570 nm. The authors need to clarify why they have used 490 nm for the MTT assay.**

***Authors response: The authors would like to extend their deepest apologies for this unintentional typological error and the correction has been done at line 255 of the revised manuscript.***

**Comment 11:** **Annexin V-FITC/PI apoptosis assay and immunoblotting of apoptosis markers would have further strengthened the results of present investigation.**

***Authors response: The authors sincerely thank the reviewer for their valuable suggestion.***

***1. The authors agree that the Annexin V-FITC/PI apoptosis assay and immunoblotting of apoptosis markers would provide more detailed and specific insights into the mechanisms of apoptosis induced by SSLE-AuNPs, further strengthening the results of our investigation. In the present study, we have provided evidence of apoptosis through nuclear fragmentation and condensation (DAPI staining), caspase-3 activation, and increased ROS generation in A549 cells treated with SSLE-AuNPs. These findings strongly suggest that SSLE-AuNPs induce apoptotic cell death in lung cancer cells.***

***2. Although the current experimentation provides strong evidence of apoptosis, we acknowledge that the inclusion of Annexin V-FITC/PI assay and immunoblotting of apoptosis markers (e.g., Bax, Bcl-2, cleaved PARP, etc.) would offer more comprehensive insights into the apoptotic pathways activated by SSLE-AuNPs. Due to resource and time constraints, these techniques were not included in the present study, but we fully recognize their importance. We fully agree with the reviewer's suggestion and plan to incorporate these techniques in our future studies.***

***3. Despite the absence of these techniques in the present investigation, the findings presented here provide a strong foundation for understanding the anticancer potential of SSLE-AuNPs. The observed nuclear fragmentation, caspase-3 activation, and ROS generation strongly support the induction of apoptosis, and we believe these results are a valuable contribution to the field.***

**Comment 12:** **Why didn’t the authors determine the cytotoxic effect of SSLE-AuNPs on J774A.1 cells? The decrease in cytokine levels is due to the anti-inflammatory activity or cytotoxicity of SSLE-AuNPs?**

***Authors response: The authors would like to acknowledge that authors have determined the cytotoxic effect of SSLE-AuNPs on J774A.1 cells. However, the results were not incorporated in the submitted manuscript and now after the concern raised by the learned reviewer, the incorporations have been made in the material and methods section at line number 251 as well as in the results sections at line number 435 to 436 (figure 5c) are included in the revised manuscript.***

***Furthermore, the authors would like to state for raising this important question regarding whether the observed decrease in cytokine levels is due to the anti-inflammatory activity of SSLE-AuNPs or their cytotoxic effects. The non-toxic concentrations of SSLE-AuNPs for the anti-inflammatory assays was carefully selected. In addition, MTT assay was performed to confirm that the concentrations used (100, 200, and 400 µg/mL) did not induce significant cytotoxicity in J774A.1 cells during the 12-hour treatment period. This ensures that the observed decrease in cytokine levels is not merely a result of cell death but reflects the true anti-inflammatory potential of SSLE-AuNPs.***

***The dose-dependent reduction in pro-inflammatory cytokines (IFN-γ and IL-1β) observed in LPS-stimulated J774A.1 cells, without significant cytotoxicity, strongly suggests that SSLE-AuNPs exert significant anti-inflammatory effects. Furthermore, the comparison between LPS-stimulated and non-stimulated cells helps isolate the anti-inflammatory activity of SSLE-AuNPs, as the reduction in cytokine levels is specific to the inflammatory response induced by LPS. The anti-inflammatory activity of SSLE-AuNPs may be attributed to their ability to modulate key signaling pathways involved in cytokine production, such as NF-κB and MAPK. Additionally, the antioxidant properties of SSLE-AuNPs, as demonstrated by their free radical scavenging activity, could contribute to their anti-inflammatory effects by reducing ROS-mediated inflammation.***

**Comment 13:** **Authors are suggested to include one inclusive section in the introduction part citing some recent references on the usage of natural compounds and green synthesized nano-formulation as an anticancer agent and their application so that the horizon of the current article could be improved further. Below articles could be covered in that recommended section:**

i. Ahmad et al. (2024) Cell Biochemistry & Function, 42: e3911

ii. Suhail et al. (2023) Frontiers in Pharmacology, 14:1236173.

iii. Ahmad et al. (2023) International Journal of Molecular Sciences, 24 (7): 6651.

iv. Suhail et al. (2023) Frontiers in Nutrition, 9: 1078642.

v. Nazam et al. (2023) Pharmaceuticals, 16: 274.

vi. Zughaibi et al. (2023) Cell Biochemistry & Function, 41: 1174-1187.

vii. Alafaleq et al. (2023) Nanomaterials, 13 (7): 1201.

viii. Alserihi et al. (2022) Nanotechnology Review, 11: 298-311.

ix. Tabrez et al. (2022) Nanotechnology Review, 11: 2714-2725.

x. Tabrez et al. (2022) Nanotechnology Review, 11: 1322-1331.

xi. Tabrez et al. (2022) Frontiers in Chemistry, 10: 970193.

***Authors response: The authors would like to sincerely thank reviewer for their valuable suggestion to include an inclusive section in the introduction discussing the usage of natural compounds and green-synthesized nano-formulations as anticancer agents. We agree that this addition will provide a broader context for our study and enhance its relevance in the field of nanomedicine and cancer therapy.***

***We have duly incorporated a new section in the introduction from line number 82 to 90 that discusses the recent advancements in the use of natural compounds and green-synthesized nano-formulations as anticancer agents. Also, this section includes recent suggested references to highlight their mechanisms of action, therapeutic potential, and applications in cancer therapy, thereby providing a stronger foundation for the current study. The stated references have been cited at reference number 2 and 8–19.***

**Reviewer 3**

The manuscript introduces interesting and valuable information on a multiple application of green-approach synthesized AuNPs. However, since the manuscript contained a lot of information, the authors must carefully check all method and discussion to control the quality of the manuscript. I believe the manuscript can be published in the journal with following modification.

**Comment 1:** **“S. splenders” should be uniformly written in Italic**

***Authors response: The authors would like to state that S. splendens has now uniformly written in italics throughout the manuscript.***

**Comment 2:** **What is “S. spp” in page 3, line 75-76 stand for?**

***Authors response: S. spp stands for Salvia species that has been revised in the manuscript in line no: 112 and page no. 5*.**

**Comment 3:** **The introduction was well arranged and written. However, I suggest that the authors should expand this section with more literature review. For e.g., based on the main content of this paper, I recommend that the authors could write a review paragraph focusing on the progress of other authors’ contribution to treating lung cancer cells. Introduction of the relationship of all the studied factors in the manuscript to lung cancer would give better comprehension to readers.**

***Authors response: As per the suggestion of learned reviewer, the authors have duly modified the introduction section of revised manuscript. The authors have included a paragraph on lung cancer from line number 91 to 101 with proper references in the revised MS.***

**Comment 4:** **Considering that the references used in this paper were only before 2024, the authors should update the references published in 2024 for “up-to-date” purpose. Combing this comment with my previous recommendation would be reasonable.**

***Authors response: The authors have updated the list of references as per articles published in 2024 in the revised manuscript.***

**Comment 5:** **In section 2.2.3, specific ratio of dried leaves and DI water (weight/volume) should be presented. In addition, if the extraction was only conducted with crushing the dried leaves with DI water at room temperature without any heating, then specific extraction time should be noted. Furthermore, practical images of DI water and leaves extract (before and after extraction) should be included in the paper for better visual and reliability.**

***Authors response: We acknowledge the reviewer’s concern regarding the need for precise experimental details. In Section 2.2.3, the aqueous extraction was performed using a standardized ratio of 1:10 (weight/volume), where 10 g of dried Salvia splendens leaves were crushed with 100 mL of double-distilled water. This ratio was selected based on preliminary optimization experiments to ensure consistent extraction efficiency of bioactive compounds. The extraction was conducted at room temperature (25 ± 2°C) without heating to prevent denaturation of heat-sensitive phytoconstituents (e.g., enzymes, polyphenols). The crushing process was performed for 15 minutes, followed by centrifugation (6,000 rpm, 15 min) to separate the supernatant. The omission of this detail in the original manuscript has been corrected, and included in revised. Furthermore, as per the insightful suggestion of learned reviewer the authors have incorporated the practical images of leaves and extract here in the rebuttal as well as in the revised manuscript also in the form of supplementary fig.1.***

**Comment 6:** **The authors used the** **terms “enzymes”, “proteins” throughout the manuscript. Due to the different of expertise, I could not confirm whether using these terms would be appropriate or not. Nonetheless, with the information the authors mentioned in the introduction section, I think “phytochemicals” would be a better and more reasonable interpretation.**

***Authors response: We sincerely appreciate the reviewer's astute observation regarding the use of "enzymes" and "proteins" versus "phytochemicals" in our manuscript. It is noteworthy to mention that we initially (2015) started working on the synthesis of gold and silver nanoparticles with plant enzymes such as bromelain and papain*** [***https://doi.org/10.1016/j.matlet.2015.06.118***](https://doi.org/10.1016/j.matlet.2015.06.118) ***due to their reported therapeutic potency. After wards, we frequently used different enzymes and drugs as reducing and capping agents for synthesizing and stabilizing inorganic nanoparticles. However, capping with protein BSA or HSA plays an important role in stabilizing inorganic nanoparticles, and we applied the same in our previous investigations. This might be the reason the terms “enzymes” and “proteins” were frequently used in the MS. However, in the present study, phytoconstituents played an important role in synthesis and capping of gold nanoparticles. Thus, we have carefully considered this suggestion and implemented the revisions in our revised manuscript.***

**Comment 7:** **In section 2.2.4.1, the authors should specifically mention how they prepared AuNPs suspension? For e.g., how would they prepare AuNPs suspension with a content of 50 µg/mL in section 2.2.5.1? Would the nanoparticles be centrifuged, dried and redispersed in water or the weight/volume were used as theoretical values (considering all Au3+ ions were converted into AuNPs)? This information should be clearly confirmed in the manuscript.**

***Authors response: We sincerely appreciate the reviewer's insightful comment regarding the preparation of the AuNPs suspension. Below is the clarification and the proposed addition to the manuscript to address this point. In this study, the AuNPs suspension was prepared by directly diluting the as-synthesized SSLE-AuNPs solution (post-synthesis purification) to the desired concentrations (e.g., 50 µg/mL) using sterile deionized water. The concentration of AuNPs was calculated based on the theoretical yield of gold (assuming complete reduction of Au³⁺ to Au⁰), after purification steps as follows:***

1. ***The initial concentration of gold (III) chloride trihydrate (HAuCl₄·3H₂O) was 1 mM (equivalent to 197 mg/L of Au).***
2. ***After 24 h of reaction with S. splendens leaf extract (SSLE), the ruby-red colloidal suspension was centrifuged at 12,000 rpm for 20 min to remove unreacted phytochemicals and salts.***
3. ***The pellet was resuspended in deionized water, and the process was repeated twice to ensure purity.***

**Comment 8:** **In page 7, line 161, should “bank” be “blank”?**

***Authors response: The authors would like to extend their deep apologies for this unintentional error and the correction has been incorporated in the revised manuscript.***

**Comment 9:** **The citation of references 15, 16, and 17 should be carefully revised by the authors, considering that the term “slight modification” would not be appropriate comparing the authors’ methods with the ones in references. As for reference 17, in this literature, there was no information about the ABTS activity assay method? If the authors still want to use these references, I suggest that these reports could be mentioned in the introduction section. Otherwise, in the response letter, the authors should clarify why those references was used and cited in those positions?**

***Authors response: We acknowledge the reviewer’s observation that the term “slight modification” may not fully capture the differences between our methods and those in references 15 and 16. To ensure accuracy, we have revised the phrasing to more precisely describe the methodological adaptations “following the general approach of [reference]. We apologize for the oversight regarding reference 17. While this citation primarily pertains to broader antioxidant evaluation principles, we recognize that it does not explicitly detail the ABTS assay. To rectify this, we have replaced it with a more directly relevant citation for the ABTS assay at reference no. 45.***

**Comment 10:** **Usually, in a ABTS assay method, K2S2O8 would be introduced to react with ABTS to produce free radical ABTS·+ cations, then the antioxidant activity of the sample was measured by the ability to convert free radical ABTS·+ cations into ABTS (monitoring by the reduction of UV-vis absorbance at 734 nm, characteristic for the existence of ABTS·+). Herein this work, according to the experimental information in section 2.2.5.2, why the authors let the AuNPs colloid react with ABTS (no free radical ions)? Why was a 50% methanolic ABTS used as blank but not a solution containing ABTS·+? The authors must carefully revise this problem because this would induce question about the reliability of this work.**

***Authors response: We sincerely appreciate the reviewer’s insightful comments regarding the ABTS assay methodology. Their expertise has helped us clarify key aspects of our experimental design. Below, we address the concerns raised and provide revisions to ensure full transparency. In our study, the pre-formed ABTS˙+ radical cation (generated by oxidizing ABTS with K₂S₂O₈) was used for the assay, as is standard practice. However, we recognize that the phrasing in Section 2.2.5.2 may have been ambiguous. To clarify this, the text has been rephrased from line number 220 to 226. We have amended the text and highlighted it in the revised manuscript to explicitly state that the assay was conducted with pre-formed ABTS˙+, not the non-radical ABTS.***

***The reviewer rightly notes that the blank should account for the ABTS˙+ radical’s absorbance. In our original submission, the term "blank" may have been misleading. To clarify this author have now replaced blank with the control containing ABTS˙+ in 50% methanol without AuNPs, ensuring the baseline absorbance reflected only the radical cation’s signal. The background correction used pure methanol (50% v/v) to account for solvent effects. We deeply value the reviewer’s rigorous critique, which has strengthened the manuscript.***

**Comment 11:** **The authors should clearly mention that ABTS was used as solution form or solid form (diammonium salt) in the Material section. In the 2.2.5.2 section, I suspect that the ABTS was directly used as commercial solution since there was no specific concentration mentioned. On the other hand, in page 15, line 318-319, ABTS was mentioned as diammonium salt. This is confusing, the authors must clarify this problem.**

***Authors response: We sincerely appreciate the reviewer’s keen observation regarding the clarification of ABTS reagent usage in our study. Below, we provide a detailed clarification to resolve any ambiguity. In our study, ABTS was used in its solid diammonium salt form (******CAS No. 30931-67-0, Sigma-Aldrich) for all experiments. The ABTS powder (≥98% purity) was precisely weighed and dissolved in ultrapure water to prepare fresh solutions for the antioxidant assays. The ABTS˙⁺ radical cation was generated in situ by reacting the solid ABTS diammonium salt with potassium persulfate (K₂S₂O₈). To improve clarity, we have revised the Methods section to explicitly state*** ***ABTS diammonium salt (7 mM) was dissolved in ultrapure water and oxidized with 2.45 mM K₂S₂O₈ to generate ABTS˙⁺. The mixture was incubated in the dark for 16 h at 25°C, then diluted with 50% methanol to an absorbance of 0.70 ± 0.02 at 734 nm (≈46.7 μM ABTS˙⁺, ε = 1.5×10⁴ M⁻¹ cm⁻¹).***

***The mention of ABTS as a "******diammonium salt" on page 15 refers to the initial solid reagent before dissolution. To avoid confusion, we have added the CAS number and supplier details in the Materials subsection (2.1) at line number 155 and specified the preparation steps in Section 2.2.5.2 to emphasize the use of solid ABTS. We thank the reviewer for highlighting this important clarification, which has improved the transparency and reproducibility of our methods. Please let us know if further details would be helpful.***

**Comment 12:** **In section 2.2.6.1, the volume of sample injected in each well should be specifically noted.**

***Authors response: We sincerely appreciate the reviewer's careful attention to methodological details. A volume of 100 μL of each sample (SSLE, SSLE-AuNPs, and tetracycline at 50μg/mL concentration) was added to the 8 mm diameter wells using a micropipette with sterile tips (line number: 235).***

**Comment 13:** **Additional analysis to confirm the formation of AuNPs (e.g. XRD, SAED, etc.) should be carried out.**

***Authors response: We sincerely appreciate the reviewer’s valuable suggestion to further characterize the biosynthesized SSLE-AuNPs using XRD and SAED. While we agree that these techniques provide deeper insights into crystallinity, we respectfully state that the existing dataset in the revised MS—combining UV-Vis spectroscopy, TEM, DLS, zeta potential and FTIR, offers robust evidence for nanoparticle formation and stability. Below, we humbly justify the importance of our current analyses and address how they collectively meet the study’s objectives:***

***1. Evidence Supporting AuNP Formation***

- ***UV-Vis Spectroscopy: The distinct surface plasmon resonance (SPR) peak at 559 nm (Fig. 1A) is characteristic of spherical AuNPs. This optical signature confirms reduction of Au³⁺ to Au⁰.***
- ***TEM Imaging: Spherical nanoparticles with an average core size of 94.8 ± 5.1 nm (Fig. 1D) were observed, ruling out aggregation or irregular morphologies.***

***2. Confirmation of Stability and Monodispersity***

- ***DLS and Zeta Potential: The hydrodynamic diameter (114.7 ± 9.8 nm) and low PDI (0.30) indicate colloidal stability (Fig. 1B). The zeta potential of −21 ± 1.9 mV (Fig. 1C) confirms electrostatic stabilization, consistent with literature thresholds for stable NPs.***

***3. Role of FTIR in establishing SSLE-AuNPs formation***

- ***FTIR Analysis: Identified the functional groups from S. splendens extract (Fig.2) that helped in reduction and capping of AuNPs. Peaks observed have shown characteristic shifts that align with mechanisms reported for plant-synthesized AuNPs.***

***4. Addressing Crystallinity Without XRD/SAED***

- ***While XRD/SAED would definitively resolve crystallographic structure, their absence does not undermine the study’s conclusions because SPR in UV-Vis is inherently linked to crystalline AuNPs. Furthermore, biological applications (e.g., antioxidant, antibacterial effects) primarily depend on size, surface chemistry (FTIR), and stability (DLS/zeta potential)—not crystallinity. Many studies on biogenic AuNPs omit XRD/SAED when focusing on bioactivity (e.g., Elia et al., Int. J. Nanomed. 2014; Katas et al., Saudi Pharm. J. 2019).***

***We acknowledge the importance of XRD for future work and will include this analysis in subsequent studies to further validate crystallinity and phase purity. However, the multifunctional biological activities of SSLE-AuNPs—demonstrated through rigorous antioxidant, antibacterial, anticancer, and anti-inflammatory assays—remain robustly supported by the existing dataset. At this point of time, we lack the facilities of XRD at our institute, and we do not have enough funds to outsource XRD analysis. We have added FTIR analysis to observe the changes in the functional groups and to confirm the attachment of phytochemicals from SSLE. We hope the reviewer agrees that the current findings provide a strong foundation for the therapeutic potential of SSLE-AuNPs, even in the absence of XRD data.***

- ***Elia P, Zach R, Hazan S, Kolusheva S, Porat ZE, Zeiri Y. Green synthesis of gold nanoparticles using plant extracts as reducing agents. International Journal of Nanomedicine. 2014;9:4007-4021. DOI: 10.2147/IJN.S57343.***
- ***Katas H, Lim CS, Azlan AY, Buang F, Busra MF. Antibacterial activity of biosynthesized gold nanoparticles using biomolecules from Lignosus rhinocerotis and chitosan. Saudi Pharmaceutical Journal. 2019;27(2):283-292.
  DOI:*** [***10.1016/j.jsps.2018.11.010***](https://doi.org/10.1016/j.jsps.2018.11.010)

**Comment 14:** **The statement in page 13, line 279-281 was suggested to not written if there was no evidence (UV-vis spectra of AuNPs colloid with different storage duration) attached in this manuscript.**

***Authors response: We appreciate the concern of the honorable reviewer, and we acknowledge that including UV-Vis spectra of the AuNPs colloid at different storage durations would have strengthened the claim in the MS. However, we found no aggregation in the SSLE-AuNPs sample even after keeping them for 6 months at room temperature (Figure). The same statement has been added to the revised MS to strengthen the claim which can also be correlated with the zeta potential and our earlier studies (as mentioned in the reply of your next comment).***

**Sample after storage at room temperature for 6 months (picture taken at the time of preparation of rebuttal showed no aggregation)**

**Comment 15:** **According to reference 32, the absolute zeta potential value needs to be higher than 30 to indicate a stable colloidal system: “Conversely, a high zeta potential (either positive or negative), typically more than 30 mV, maintains a stable system”. Even though the comment on the zeta potential for a stable colloidal system would be controversial, it was commonly concluded that an absolute zeta potential higher than 30 mV would be corresponding to a stable colloidal system (Surendra Nimesh, Ramesh Chandra, Nidhi Gupta, Advances in Nanomedicine for the Delivery of Therapeutic Nucleic Acids, 2017; https://doi.org/10.1002/cjce.23914). Hence, the discussion stating that the AuNPs colloidal system having a zeta potential value of 21 mV was stable should be carefully reclaimed.**

***Authors response: We appreciate the concern of the reviewer; however, we would most humbly bring into kind notice of the reviewer that our team has been working on metallic nanoparticles synthesis since last decade and developed several methods for their synthesis. We observed from our experience in the field of nanoparticle synthesis that specifically metallic nanoparticles even though the zeta is -18mV, it remains quite stable for months without aggregation if capping is perfectly done [1-13]. However, a capping agent plays an important role in deciding the zeta potential and stability of nanoparticles. Here, the claim was not only based on the zeta value, but it also depends on the aggregation ability after storage and our previous experiences.***

***However, we have modified our discussion to more precisely reflect the stability characteristics. The measured zeta potential of -21 ± 1.9 mV suggests moderate colloidal stability, as values between ±20-30 mV typically indicate the onset of stability (DoI: 10.4314/tjpr.v12i2.20). The corrections “confirming relative colloidal stability of the synthesized nanoparticles” have been included at line number 350 to 352 of the revised manuscript. While absolute values >30 mV are considered optimal for long-term stability (Nimesh et al., 2017), our system showed no visible aggregation even after six months, likely due to synergistic stabilization from both electrostatic repulsion and steric effects of the plant-derived capping agents.***

1. Lila AS, Moin A, **Rizvi SM**. Synergy of moxifloxacin loaded gold nanoparticles and resveratrol against ocular pathogens: a combinatorial approach against eye infections. Journal of Drug Delivery Science and Technology. 2025 Mar 17:106823.
2. Abu Lila A, Awadalkarim AM, AL-Shammary AA, Jayaramu RA, Moin A, Ahmad N, SB S, Gopalpura PB, **Rizvi SM**. Eco-friendly synthesis, characterization and biological evaluation of silver nanoparticles using Ylang-Ylang Oil and Tea-Tree Oil. Nano Express. 2024 Dec 1.
3. Al Hagbani T, **Rizvi SM**, Shakil S, Lila AS. Nano-Formulating Besifloxacin and Employing Quercetin as a Synergizer to Enhance the Potency of Besifloxacin against Pathogenic Bacterial Strains: A Nano-Synergistic Approach. Nanomaterials. 2023 Jul 16;13(14):2083.
4. **Rizvi SM**, Hussain T, Alshammari F, Sonbol H, Ahmad N, et al. Nano-Conversion of Ineffective Cephalosporins into Potent One against Resistant Clinical Uro-Pathogens via Gold Nanoparticles. Nanomaterials. 2023; 13(3):475. https://doi.org/10.3390/nano13030475
5. Alafnan A, **Rizvi SM**, Alshammari AS, Faiyaz SSM, Lila ASA, Katamesh AA, et al. Gold Nanoparticle-Based Resuscitation of Cefoxitin against Clinical Pathogens: A Nano-Antibiotic Strategy to Overcome Resistance. *Nanomaterials*. 2022; 12(20):3643. https://doi.org/10.3390/nano12203643
6. Al Hagbani T, **Rizvi SM**, Hussain T, Mehmood K, Rafi Z, Moin A, Abu Lila AS, et al. Cefotaxime Mediated Synthesis of Gold Nanoparticles: Characterization and Antibacterial Activity. *Polymers*. 2022; 14(4):771. https://doi.org/10.3390/polym14040771
7. Alshammari F, Alshammari B, Moin A, Alamri A, Al Hagbani T, Alobaida A, Baker A, Khan S, **Rizvi SM**. Ceftriaxone Mediated Synthesized Gold Nanoparticles: A Nano-Therapeutic Tool to Target Bacterial Resistance. *Pharmaceutics*. 2021; 13(11):1896.
8. Soliman WE, Khan S, **Rizvi SM**, Moin A, Elsewedy HS, Abulila AS, Shehata TM. Therapeutic Applications of Biostable Silver Nanoparticles Synthesized Using Peel Extract of Benincasa hispida: Antibacterial and Anticancer Activities. Nanomaterials (Basel). 2020 Sep 30;10(10):1954.
9. Shaikh, S., Nazam, N., **Rizvi, S.M**., Ahmad, K., Baig, M.H. et al. Mechanistic insights into the antimicrobial actions of metallic nanoparticles and their implications for multidrug resistance. Int J Mol Sci. 2019 May; 20(10): 2468.
10. Khatoon, A., Khan, F., Ahmad, N., Shaikh, S.,**Rizvi, S.M**., Shakil, S., et al. 2018. Silver nanoparticles from leaf extract of Menthapiperita: Eco-friendly synthesis and effect on acetylcholinesterase activity. Life Sci. 209: 430-434.
11. **Rizvi, S.M**., Hussain, T., Ahmed, A.B.F., Alshammari, T.M. et al. 2018. Gold nanoparticles: A plausible tool to combat neurological bacterial infections in humans. Biomed Pharmacother. 107:7-18.
12. Shaikh S., **Rizvi S.M**., Shakil S., Hussain T., Alshammari T.M., Ahmad W., Tabrez S.,Al-Qahtani M.H., Abuzenadah A.M. 2017. Synthesis and Characterization of Cefotaxime Conjugated Gold Nanoparticles and Their Use to Target Drug-resistant CTX-M-producing Bacterial Pathogens. *J Cell Biochem*.118(9): 2802-2808.
13. Khan S., **Rizvi S.M**., Avaish M., Arshad M. et al. 2015, A novel process for size controlled biosynthesis of gold nanoparticles using bromelain. *Materials Letters*159: 373–376.

**Comment 16:** **How the authors analyzed the average particle size from TEM image (Fig. 1D)? As the Fig. 1D illustrated, the largest particle diameter would be around 80 nm, which indicated that the average size would be a value that is smaller than 80 nm. The authors can consider use another TEM image with larger scale to observe more particles, which might induce better average particle size value.**

***Authors response: The manuscript has been updated to include a particle size distribution graph (figure 1e) alongside the revised TEM image (figure 1d). The particle size distribution graph details have been duly added in the revised MS.***

**Comment 17:** **In addition, particle size distribution graph should be included together with TEM image. From this analysis, the difference or the relationship between average particle size obtained from TEM image and DLS analysis should be discussed.**

***Authors response: We acknowledge the concern of the reviewer; we have duly added the particle size graph with TEM image (Figure 1e) along with DLS z-average size (Figure 1b). However, particle size distribution graph of TEM showed the average size as 90.78 nm, while DLS Z-average particle size was 114.7 nm. The differences in average size by both the techniques is due to the different principles applied for the measurement of size.***

***The DLS graph illustrates Z-average particle size which shows monomodal size distribution based on the hydrodynamic diameter distribution of SSLE-AuNPs with a polydispersity index (PDI) of 0.30, that confirms moderate homogeneity. DLS hydrodynamic diameter of 114.7 ± 9.8 nm includes information of the core, phytochemical capping layer, and solvation shell. On the other hand, TEM Analysis revealed an average core size of 90.78 nm for SSLE-AuNPs, measuring only the size of inorganic gold core. In short, the larger size from DLS is attributed to the dynamic movement of nanoparticles in solution, where the hydrated capping layer (composed of S. splendens phytochemicals) and solvent molecules contribute to the apparent size. In contrast, TEM measures dry, static particles under vacuum. DLS overestimates size for polydisperse samples due to its sensitivity to larger particles and TEM provides precise core dimensions but may miss the organic corona visible in solution. Thus, both the strategies are important for the analysis of nanoparticle samples. Importantly, we have got same pattern in our previous investigations on gold nanoparticles. We have duly highlighted the statement regarding these size differences in the revised MS.***

**Comment 18:** **p-value is used for hypothesis testing in statistic study. Therefore, p-value should not be used in this work to interpret for average value of 3 measurements.**

***Authors response: The authors would like to bring in kind consideration of learned reviewer that in the present work, p value was used to interpret the statistical significance between groups and control in anticancer studies. Furthermore, the level of significance during stated comparison was also determined based on p value using one Anova and post hoc test. Therefore, the authors believe that omitting the p value from the manuscript will hamper the scientific interpretation of anticancer results. Considering the importance of p-value for anticancer outcomes, we request reviewer to keep it as it is in the MS.***

**Comment 19:** **Based on the results studying DPPH and ABTS assays, IC50 values of SSLE-AuNPs (µg/mL) could be determined and compared with IC50 value of ascorbic acid (standard in authors’ study). This result could be more valuable and replaced for simple statement of “The SSLE-AuNPs exhibited a dose dependent anti-oxidant activity as manifested by substantial neutralization of DPPH and ABTS radicals” in the abstract.**

***Authors response: We sincerely appreciate the reviewer’s insightful suggestion to quantify the antioxidant efficacy of SSLE-AuNPs through IC50 values in comparison to ascorbic acid (the standard antioxidant). The initial focus was on relative scavenging percentages (Table 1) to highlight dose-dependency. However, we agree that IC50 values provide a standardized metric for cross-study comparisons. As recommended, we have now included the comparative IC50 values of SSLE-AuNPs and ascorbic acid (standard) for both DPPH and ABTS assays in the revised abstract (highlighted for ease of reviewer line no. 44–46).***

**Comment 20:** **If the MIC50 values corresponding to the antibacterial activity were determined from the graphs in Figure 3, then the graphs should be illustrated in form of line + symbol instead of curve + symbol.**

***Authors response: We thank the reviewer for this valuable technical suggestion regarding the data presentation in Figure 3. As per the suggestion the authors have incorporated graphs (fig. 3c) in the revised manuscript illustrated in form of line + symbol instead of curve + symbol and added grid lines to help in finding the MIC50.***

**Comment 21:** **If the authors aimed to provide information about the as-synthesized AuNPs for medicinal purpose (treating lung cancer cells), then the cytotoxicity of SSLE-AuNPs on human cells should also be studied. A limit of using AuNPs concentration could help authors determined specific parameters in lung cancer cells treating, instead of just introducing relationship of colloidal concentration to the viability assay study.**

***Authors response: We sincerely appreciate the reviewer’s insightful suggestion regarding the importance of evaluating the cytotoxicity of SSLE-AuNPs on human cells for medicinal applications. The reviewer rightly highlights the need to establish safe concentration limits to optimize therapeutic efficacy while minimizing off-target effects. Nevertheless, to prove the cytotoxicity of synthesized SSLE-AuNPs, the authors have tested the cytotoxicity of synthesized SSLE-AuNPs against normal murine alveolar macrophages (J774A.1) cells. The same has been added in the revised manuscript and highlighted in the material and methods as well as result section (fig. 5c).***

**Comment 22:** **In the cytotoxicity assay study on lung cancer cells of SSLE-AuNPs, authors should provide the cell viability of SSLE sample (without AuNPs).**

***Authors response: We thank the reviewer for this important suggestion. While our original study focused on SSLE-AuNPs' bioactivity, we acknowledge that including SSLE-only cytotoxicity data would provide valuable comparative information. We have now conducted additional MTT assays with SSLE alone (without AuNPs) under identical experimental conditions (24h exposure, same concentration range: 50-400μg/mL) and the same has been included in the revised manuscript (fig. 5b).***

**Comment 23:** **The error line in graphs should be illustrated with black color.**

***Authors response: The authors have incorporated the suggested correction in the revised manuscript.***

**Comment 24:** **There are spelling mistakes in the manuscript, the authors should carefully check the manuscript again.**

***Authors response: As per the suggestion of learned reviewer, the authors have checked the manuscript for spelling mistakes within the revised manuscript.***

**Comment 25:** **The images’ quality (resolution) should be improved for better visual.**

***Authors response: The authors have improved the resolution of images in the revised manuscript.***

**Comment 26:** **The results in Table 2 and 3 should interpreted in form of graphs for better comparison and visualization.**

***Authors response: As per the suggestion of learned reviewer, the authors have now interpreted data of table 2 and 3 in the form of graphs (fig. 3c and 4c) in the revised manuscript.***

**Reviewer 4**

**The article is nicely written and easily understandable.**

**Comment 1:** **It would be better if the authors clarified the functional compounds that were responsible for reducing the agent from Au+ to Au in their study. By providing specific details about these compounds, readers would gain a clearer understanding of the mechanisms at play and could more effectively evaluate the implications of the research. Otherwise, the article is understandable.**

***Authors response: We sincerely thank the reviewer for raising this important point. While our study did not perform HPLC or characterization of extract, however we can clarify the reduction mechanism based on the following evidence from our existing experimental data and cited literature.***

1. ***UV-Vis Kinetics: The rapid reduction of Au³⁺ (complete within 24 h at 40°C) suggests reduction by phytochemicals present in SSLE, as we have not added any extra reducing or capping agent during synthesis.***
2. ***Zeta Potential & Stability: The negative zeta potential (−21 ± 1.9 mV) indicates successful capping and stabilization of SSLE-AuNPs by the phytochemicals present in SSLE.***
3. ***We have added FTIR that confirms the successful attachment of functional group present in SSLE onto AuNPs.***
4. ***The reduction is attributed to redox-active phytochemicals in SSLE (e.g., anthraquinones, flavonoids), consistent with established S. splendens composition (Chopra et al., 2015).***

***Our focus was to synthesize SSLE-AuNPs with biomedical potential and we have used the similar approach in the past for silver nanoparticles [1-3]. However, we can proceed with deciphering the components of SSLE responsible for the synthesis in our future investigations. However, the authors have added paragraphs pertinent to plausible green synthesis mechanisms (based on literature) and limitations of the study in the revised MS.***

1. Soliman WE, Khan S, **Rizvi SM**, Moin A, Elsewedy HS, Abulila AS, Shehata TM. Therapeutic Applications of Biostable Silver Nanoparticles Synthesized Using Peel Extract of Benincasa hispida: Antibacterial and Anticancer Activities. Nanomaterials (Basel). 2020 Sep 30;10(10):1954.
2. Khatoon, A., Khan, F., Ahmad, N., Shaikh, S.,**Rizvi, S.M**., Shakil, S., et al. 2018. Silver nanoparticles from leaf extract of Menthapiperita: Eco-friendly synthesis and effect on acetylcholinesterase activity. Life Sci. 209: 430-434.
3. Abu Lila A, Awadalkarim AM, AL-Shammary AA, Jayaramu RA, Moin A, Ahmad N, SB S, Gopalpura PB, **Rizvi SM**. Eco-friendly synthesis, characterization and biological evaluation of silver nanoparticles using Ylang-Ylang Oil and Tea-Tree Oil. Nano Express. 2024 Dec 1.
